# Application Prospects of a Silicon-Based MEMS Safety and Arming Device for a Micro-Explosive Train

**DOI:** 10.3390/mi16050497

**Published:** 2025-04-24

**Authors:** Wei Ren, Dongpeng Zhang, Enyi Chu, Tengjiang Hu, Anmin Yang, Hui Li, Jianhua Chen, Jiao Li, Wei Liu

**Affiliations:** 1 State Key Laboratory of Transient Chemical Effects and Control, Shaanxi Applied Physics and Chemistry Research Institute, Xi’an 710061, China; 2 State Key Laboratory for Manufacturing System Engineering, Xi’an Jiaotong University, Xi’an 710049, China

**Keywords:** pyrotechnic, safety and arming device, micro-explosive train, MEMS

## Abstract

As the initial energetic device and driving force of weapon systems, pyrotechnics serve as the core and most sensitive explosive initiating device of weaponry. To accommodate the development requirements of various informatized and miniaturized weapons, MEMS pyrotechnics, characterized primarily by energy conversion informatization, structural miniaturization, and train integration, have become a significant direction in the development of pyrotechnics technology. MEMS Safety and Arming Devices, serving as the energy transfer control mechanisms for micro-explosive trains in MEMS pyrotechnics, are one of the key technologies in the design of MEMS pyrotechnics. This study conducted a classification study of a silicon-based MEMS Safety and Arming Device from the perspective of micro-explosive train structures, analyzed the technical principles of different S&A device, explored their application progress and research status, and summarizes the trends of the micro-miniaturization, integration, and informatization of the silicon-based MEMS Safety and Arming Device, providing new ideas for the research and the design of MEMS Safety and Arming Devices.

## 1. Introduction

Pyrotechnics is a general term for disposable devices or systems that convert firing control information into firing energy, stimulate chemical reactions in energetic materials, and release combustion, explosive energy, or mechanical energy in a specific train to achieve ignition/detonating/work [1]. As the core unit and the most sensitive explosive initiating unit of weaponry, pyrotechnics are widely used in six fields: fuze-warhead damage, launch power, aerospace vehicles, missile and rocket structures, passive jamming equipment, and non-combat/industrial equipment. Their safety and reliability directly affect the effectiveness of weaponry [2]. Driven by the demands of miniaturization and informatization in weapon systems, the primary development direction of pyrotechnics is the fourth generation, characterized by energy conversion informatization, structural miniaturization, and train integration. MEMS pyrotechnics are a prime example of the fourth generation and represent a significant direction in the technological development of pyrotechnics [3].

**Figure 1:**
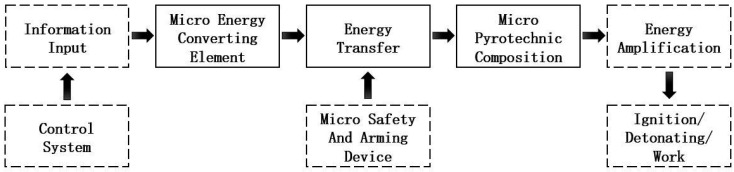
Schematic diagram of a micro-explosive train.

MEMS pyrotechnics utilize MEMS manufacturing technology to integrate three key components: a micro-energy-converting element, micro-pyrotechnic composition, and micro-safety and arming device onto a micro-substrate, forming a micro-explosive train that is integrated and packaged with control chips, achieving the miniaturization and integration of MEMS pyrotechnics. In order to ensure the safety and reliability of the micro-explosive train, MEMS pyrotechnics are equipped with safety and arming devices between the micro-energy-converting element and micro-pyrotechnic composition, which achieve the functions of explosion isolation and transmission through the action of the micro S&A device. The working principle of the micro-explosive train is shown in Figure 1, where energy transfer is controlled by the micro S&A device.

Regarding the material selection for MEMS S&A devices, there are mainly two categories: non silicon-based (usually metal based) and silicon-based materials. Non silicon-based MEMS S&A devices can achieve the function of releasing insurance by sensing changes in environmental forces. However, previous non-silicon based MEMS manufacturing processes were not mature, resulting in large volumes and low levels of intelligent control of non-silicon-based S&A devices, which cannot meet the development needs of modern weapon systems for miniaturization and intelligence. And silicon-based MEMS S&A devices, such as electrically driven silicon-based MEMS S&A devices, are easy to input and control electrical signals. By using an electrically driven S&A device to release the fuse, the passive release method through environmental forces is transformed into an active-release method controlled by electrical signals, which has stronger design scalability. Silicon-based MEMS S&A devices have good compatibility with microelectronic processing technology, and can achieve integrated design and manufacturing with MEMS pyrotechnics, adding information recognition and firing control functions to MEMS pyrotechnics in complex combat environments, further promoting the development of MEMS pyrotechnics transduction informatization, structural miniaturization, and train integration.

This paper classifies and studies a silicon-based MEMS S&A device based on micro-explosive train structures, aiming to comprehensively explore the application research progress of the silicon-based S&A device for micro-explosive trains, and analyze their technical principles, development status, challenges faced, and future trends.

## 2. Research Status of MEMS S&A Devices

MEMS S&A devices are mainly used to control the transmission of signals and energy in MEMS pyrotechnic devices, playing a key role in the control of signal and energy transmission in explosion sequences. Their most basic functions can be summarized as safe and reliable [4,5,6]. Safety refers to the ability of a MEMS pyrotechnic device to avoid accidents during use, storage, transportation, and other processes, ensuring the safety of personnel and equipment. The safety indicators of MEMS pyrotechnic devices require that the MEMS actuator can only be released under predetermined conditions and must not be released under any other circumstances. Reliability refers to the ability of MEMS pyrotechnic devices to complete their predetermined functions under specified conditions and within a specified time. The reliability index of MEMS pyrotechnic devices requires that the MEMS actuator must complete its function in a predetermined manner, mainly including the reliability of the insurance state, the reliability of releasing the insurance, and the reliability of energy transfer.

### 2.1. Classification of Micro-Explosive Trains

There are mainly two types of train structures for micro-explosive trains: displaced micro-explosive trains and isolated micro-explosive trains [7].

**Figure 2:**
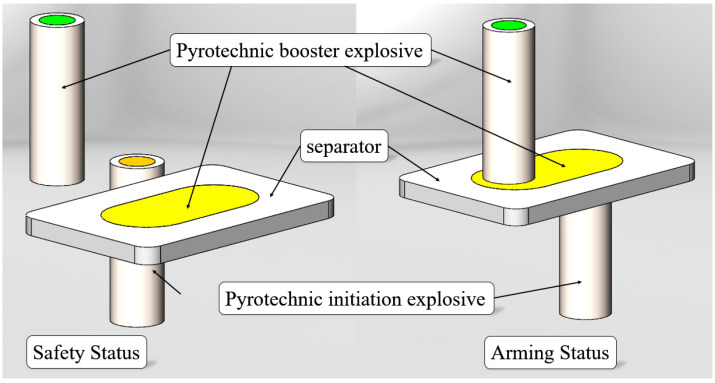
Schematic diagram of a displaced micro-explosive train.

As shown in Figure 2, the displaced micro-explosive train is a method of arranging the energy transfer routes of the explosive train in a displaced manner. The energy transfer is mainly achieved by transferring the detonation energy, resulting in a larger overall output energy. The working principle is as follows: the energetic agent is arranged inside the sliding plate and placed in a displaced position with the energetic agent in the energy amplification mechanism. During service work, due to the misalignment of the energy transmission route, the safety of MEMS pyrotechnics is ensured; when the weapon approaches the target, the driver pushes the sliding plate to move, completing the alignment of the energetic agent in the train. At this time, the MEMS pyrotechnics is in a released state.

**Figure 3:**
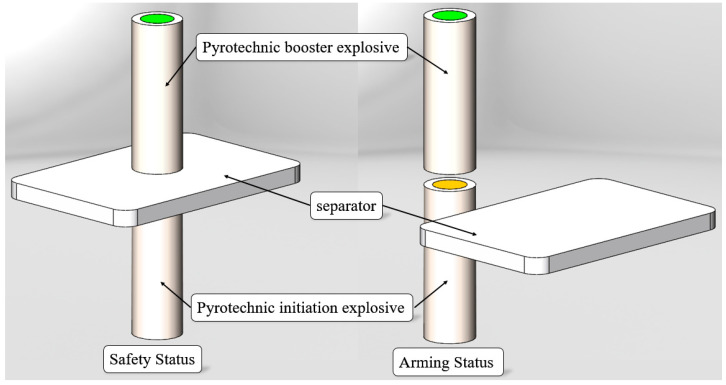
Schematic diagram of an isolated micro-explosive train.

As shown in Figure 3, the isolated micro-explosive train arranges the energy transmission route of the explosive train in the same straight-line direction, and its energy transmission can be achieved by high-speed flyer impact or laser detonation of energetic agents. The overall structure is relatively compact. Its working principle is as follows: the partition is placed between the energetic agents. During duty work due to the obstruction of the partition, the energy transfer channel in the train is closed, ensuring the safety of the weapon system; when the weapon approaches the target, the driver pushes the partition to move, the energy transfer channel in the train opens, and the energetic material can be excited. At this time, the MEMS pyrotechnics is in a protected state.

Traditional S&A devices often adopt a displaced micro-explosive train structure. Due to the limitation of displacement, the overall size of the device is difficult to reduce. In addition, energetic materials are introduced into each part of the device structure, making it difficult to be compatible with MEMS processes [8,9,10,11]. In recent years, a large amount of research has been conducted on the in situ growth of micro-pyrotechnic compositions both domestically and internationally. Although the micro-assembly problem between agents and structures has been preliminarily solved, there is still a certain distance to be covered in applying it to the integration of microsystems. The MEMS S&A device structure using an isolated micro-explosive train structure is compact, which is conducive to the overall miniaturization of the system. Its detonation method can be adapted to various pyrotechnic devices, such as impact plate detonators and laser detonators, with lower requirements for assembly accuracy between structures, making it easier to achieve system structural design and manufacturing.

### 2.2. Displaced Explosive Train Structure MEMS S&A Device

**Figure 4:**
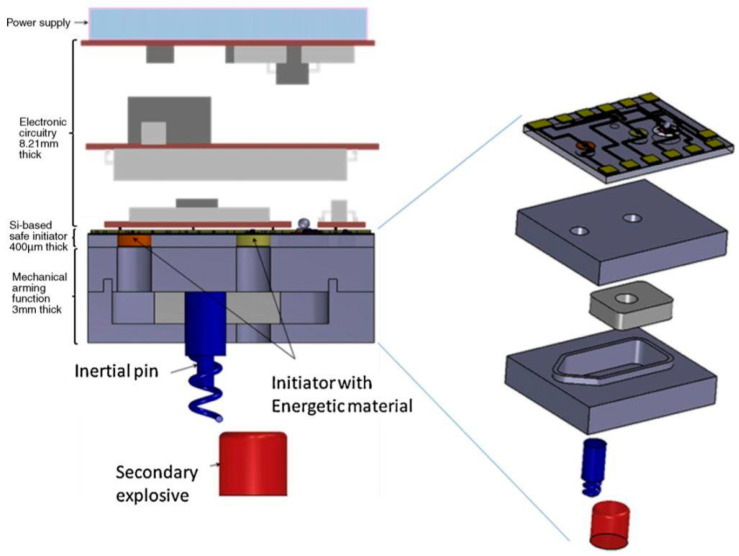
Pyro MEMS S&A device. Reprinted from Ref. [14].

Hu Tengjiang elaborated on the current situation of displaced explosive train structure MEMS S&A devices in his paper [12]. Helene Pezous from the French Centre for Scientific Research (CNRS) reported in 2010 on the MEMS S&A device she developed [13,14], as shown in Figure 4. The MEMS S&A device is driven by the principle of fireworks. During the flight of the weapon, the corresponding environmental forces (such as centrifugal force) will cause the inertial locking pin and partition to unlock. At this time, the electrical signal controls the detonator to complete firing, and the generated high-temperature and high-pressure gas will push the partition to move, achieving the overall device release. The overall energy consumption of the MEMS S&A device is only 635 mW, and the overall size is less than 10 mm × 10 mm × 10 mm.

**Figure 5:**
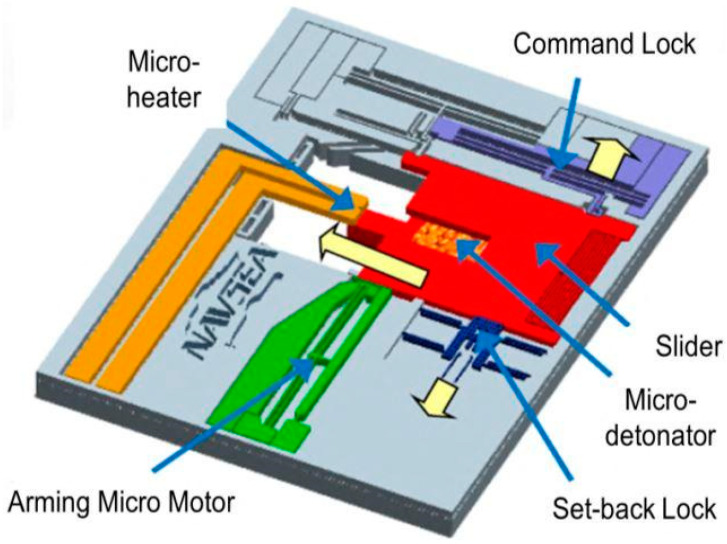
Electrothermal mechanical MEMS S&A device. Reprinted from Ref. [15].

Taylor T. Young from the US Navy Surface Warfare Center reported on a silicon-based MEMS S&A device at the 2016 Fuze Annual Meeting [15], with its specific structure shown in Figure 5. In a safe state, the igniter is placed in a displaced position with the explosive in the sliding partition, and the mechanism is locked by the command lock, recoil lock, and micro-driver to ensure the safety of the weapon during storage and transportation. When the MEMS S&A device is released, the rear seat lock first acts, and the device achieves primary release. Then, under the control of the missile-mounted sensor, the electric micro-driver and the command lock successively release the constraints on the sliding partition. Under the action of the weapon rotation acceleration, the sliding partition will align with the igniter, and at this time, the device completes the release action. At present, this silicon-based MEMS S&A device has started relevant performance testing experiments on 40 mm caliber grenades.

**Figure 6:**
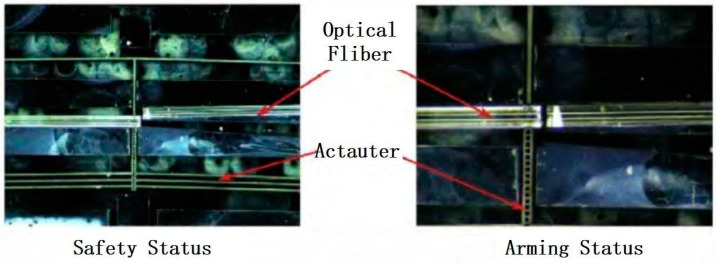
Laser-ignition MEMS S&A device. Reprinted from Ref. [16].

Zhou Xiaodong from the Army Engineering University of the People’s Liberation Army of China proposed a laser-ignited MEMS S&A device in 2017 [16], as shown in Figure 6. Two optical fibers are misplaced in the mechanism. When the device receives the execution signal, the silicon-based electric S&A device starts to operate, completing the mutual alignment of the two optical fibers. At this time, laser energy can be transmitted normally and ignite the next level of energetic material. The device can achieve mutual conversion between safety and release states within 19 ms, while the overall size is less than 10 mm × 10 mm × 0.5 mm.

### 2.3. Isolated Explosive Train Structure MEMS S&A Device

**Figure 7:**
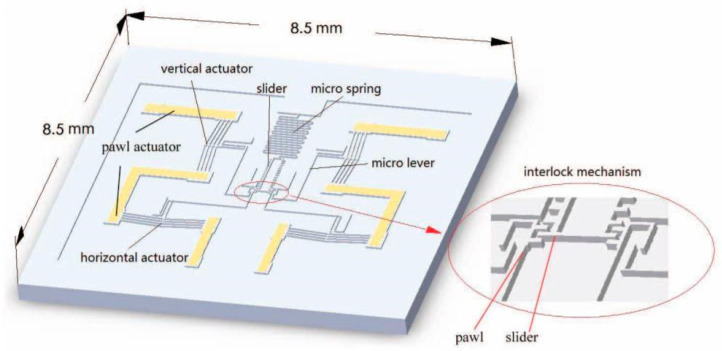
Large displaced electrothermal MEMS S&A device. Reprinted from Ref. [17].

Hu Tengjiang from Xi’an Jiaotong University proposed a large displaced MEMS S&A device in 2019 [17], as shown in Figure 7. Two claws that can drive the slider separately are symmetrically placed in the mechanism, consisting of a horizontal electric S&A device and a vertical electric S&A device. The horizontal electric S&A device is used to pull the slider. The vertical electric S&A device is used to achieve engagement and separation between the pawl and the slider. The teeth on the pawl and slider can not only achieve interlocking function, but also achieve linear displacement during operation. The MEMS S&A device switches between safety and release states by driving the partition to achieve linear displacement. The size of the electrically driven MEMS S&A device is 8.5 mm × 8.5 mm × 0.8 mm.

**Figure 8:**
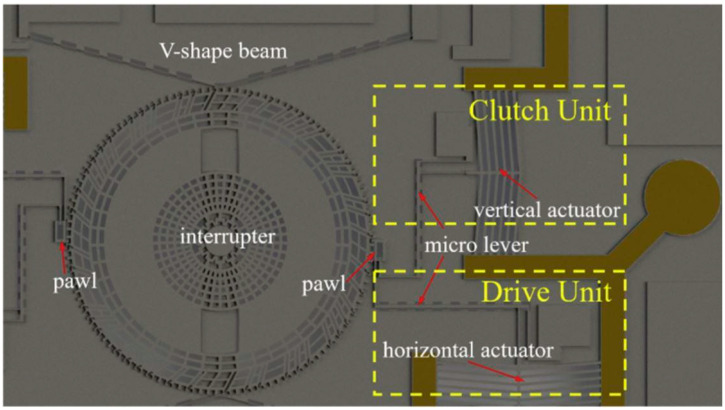
MEMS S&A device with an encryption function. Reprinted from Ref. [18].

Fang Kuang from the Institute of Chemical Materials at the Chinese Academy of Engineering Physics proposed a MEMS S&A device with encryption function driven by a V-shaped electric micro S&A device in 2020 [18], as shown in Figure 8. The control unit of the S&A device consists of a vertical electric S&A device and a micro-lever, used to control the separation and engagement between the pawl and the partition plate. The driving unit consists of a horizontal electric S&A device and a micro-lever, which is used to pull the partition plate to rotate on the substrate. The V-shaped beam can be wedged into the tooth groove between the teeth on the partition plate to fix the position of the partition plate. The teeth of the turntable are divided into normal teeth and trap teeth, and the pawl is equipped with a unique locking groove. When an incorrect decoding voltage is input, it can form a bite lock with the spring in the trap teeth, and the entire device is locked. The safety mechanism switches between safety and release states by controlling the rotation of the partition plate through a pawl. The overall size of the device is 13.4 mm × 9.3 mm × 0.454 mm.

### 2.4. Analysis of the Current Situation of MEMS S&A Devices

As shown in Table 1, MEMS S&A devices used for micro-explosion sequences can be divided into displaced explosive train structure MEMS S&A devices and isolated explosive train structure MEMS S&A devices according to the explosion sequence. According to the manufacturing materials, it can be divided into silicon-based MEMS S&A devices and non silicon-based MEMS S&A devices (mostly metal-based MEMS S&A devices). Non silicon-based actuators are generally larger in size and have poor compatibility with MEMS processes, but their structural strength is relatively high, while silicon-based actuators have better compatibility with MEMS processes and stronger design scalability. According to the different driving methods of micro-actuation mechanisms, MEMS S&A devices can be divided into environmental force driving, gunpowder force driving, electric thermal force driving, electromagnetic force driving, piezoelectric principle driving, shape memory alloy driving, and multi-principle joint driving. Kan Wenxing et al. summarized and organized the driving principles, driving conditions, output efficiency, application platforms, and other aspects of MEMS S&A devices with different driving methods [19].

## 3. Prospects

### 3.1. Miniaturization and Integration

The integration of MEMS S&A devices cannot be separated from the modular design concept of fuses [20]. The concept of a modular design for fuses emerged in the 1990s, dividing fuse systems into safety control, firing control, explosive train, energy system, and other modules based on their functions. Each module is designed according to the overall design requirements and technical characteristics of the fuse, and structurally independent modules are produced using methods such as mechanical processing. The functional modules are combined into a fuse system through interfaces and wire connections. MEMS pyrotechnics with built-in safety devices integrate MEMS S&A devices with MEMS pyrotechnics to achieve safety control at the pyrotechnic level. By actively controlling the action of the insurance mechanism through electrical signals, the isolation and alignment of micro-detonation trains can be achieved, effectively improving the safety and reliability of pyrotechnic devices.

The key to achieving the miniaturization and integration design of MEMS S&A devices and MEMS pyrotechnics through the combination of microscale detonation train technology, MEMS technology, and advanced micro-mechanical processing technology lies in the breakthrough of MEMS manufacturing processes.

#### 3.1.1. Composite Materials for MEMS S&A Device Manufacturing

At present, MEMS S&A devices mainly use silicon materials to achieve corresponding energy isolation functions. Compared with metal or ceramic materials, silicon separators will break after the impact of flying blades. Although they can ensure that the detonating charge column at the back end is not excited, its reliability cannot be characterized. Therefore, conducting research on composite-based MEMS S&A devices will become a future focus of work. Taking metal as an example, the common high-strength partition materials are Ni or Cu, and a thickness of 200 μm can ensure that they are not shattered by metal flying pieces. Considering that metal materials make it difficult to achieve high-precision tooth shape processing, a combination of MEMS technology and traditional mechanical processing technology can be used to produce high-precision silicon partitions with tooth groove structures and metal partitions without tooth groove structures, respectively. The two can be aligned and fixed in corresponding positions through a micro-assembly platform. The driver is made of a silicon material and assembled with a metal–silicon-based composite separator and a silicon-based driver under the operation of a micro-assembly platform.

**Figure 9:**
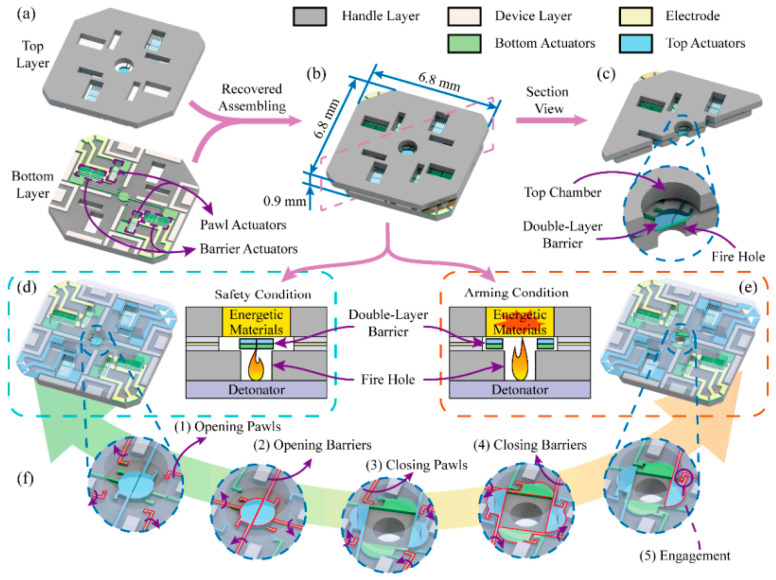
Metal/silicon composite barrier. (**a**) Two layers structure; (**b**) The S&A device; (**c**) The section view; (**d**) The safety condition; (**e**) The arming condition; (**f**) Operation process of the bistable mechanism. Reprinted from Ref. [21].

As shown in the Figure 9, Wang Kexin has introduced a novel MEMS S&A device with the metal/silicon composite barrier in order to enhance the fabrication precision and the structural strength of a MEMS S&A (Micro-Electro-Mechanical System Safety and Arming) device simultaneously [21].

#### 3.1.2. Precision of the MEMS S&A Device Structure

The manufacturing method of MEMS S&A devices has gradually shifted from LIGA technology to silicon technology, and now to metal-based MEMS processing, laser processing technology, etc., making it possible to manufacture devices with a smaller volume, more complex structure, and highly integrated functions. Through an integrated design, detection, driving, explosion-proof, and explosion transmission functions are integrated, and the output energy density of micro-energetic materials is much higher than that of traditional macro-energetic materials. Therefore, MEMS S&A devices are required to provide larger output displacement (greater than micrometers) to ensure the overall safety performance of the device.

**Figure 10:**
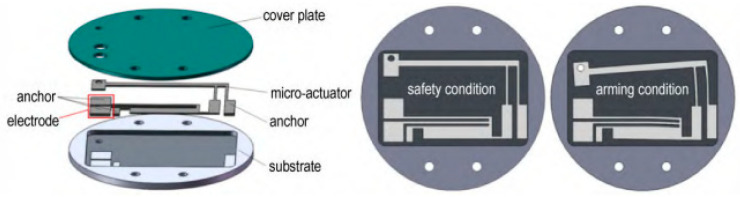
The structure of the safety and arming device. Reprinted from Ref. [22].

As shown in Figure 10, Zhang Yun researched and designed a metal material electric heating-driven micro-safety mechanism based on laser processing. Finite element simulation was used to analyze the action characteristics of nickel-based micro-electric actuators. By adjusting the length, width, and gap between the hot and cold arms of the double-hot-arm U-shaped electric actuator, their effects on output displacement were explored. Finally, a performance verification platform for security institutions was established to verify their driving displacement and explosion-proof performance under different current excitations [22].

#### 3.1.3. Diversified Driving Methods for MEMS S&A Devices

Early MEMS S&A devices could only be driven in specific inertial environments, and this passive driving method makes it difficult to meet the requirements of the current complex battlefield environment. MEMS S&A devices can comprehensively meet the development needs of next-generation weapon systems intelligence by combining environmental force driving with active driving (such as electric heating driving, electromagnetic driving, etc.). In complex battlefield environments, simple active driving methods such as electric heating or electromagnetic driving may face safety and functional challenges due to environmental disturbances. For example, strong electromagnetic interference can significantly reduce the reliability of electromagnetic drives. In order to meet the high standards of safety and reliability required by modern weapons and equipment, MEMS S&A devices must integrate both environmental-force-driven and active-drive mechanisms simultaneously. This composite driving strategy not only enhances the adaptability of the system, but also ensures stability and efficient operation under various extreme conditions. Therefore, developing diversified driving methods or improving the anti-interference ability of passive and active driving methods for specific environments can effectively enhance the environmental adaptability and practicality of MEMS S&A devices.

**Figure 11:**
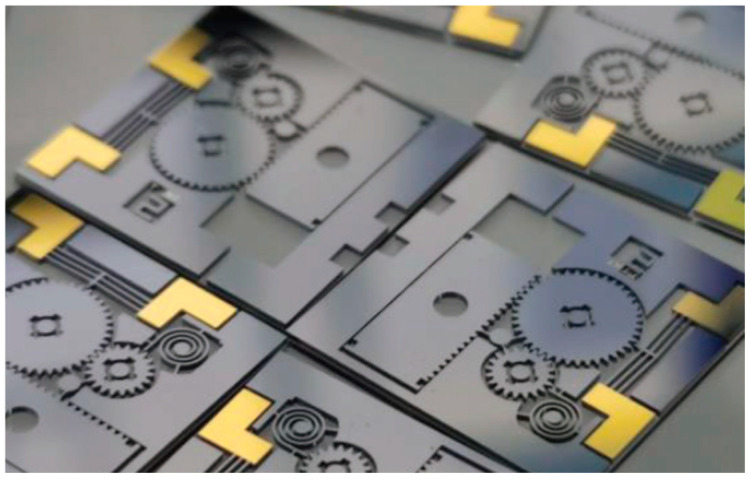
Overall structure of a MEMS actuator. Reprinted from Ref. [23].

As shown in Figure 11, Li Hui released a MEMS actuator from the dual environment designed through research on the driving mechanism and structure design of a MEMS silicon-based actuator that can realize the dual-environment force release function. It is based on the mass-gear mechanism (inertial drive) centrifugal force drive design [23]. The actuator can achieve a 1.5 mm output displacement under the control of 10 V DC voltage. Under the condition that the centrifugal is not less than 5000 r/min, it can realize the safety release function, and can resist overload 10,000 g. The micro-detonation device has a high degree of integration, initially realizes the function of releasing the force of the silicon-based inertial environment, and has good compatibility with MEMS technology.

#### 3.1.4. MEMS S&A Device Partition Self-Recovery Function

The current MEMS S&A device does not have a self-recovery function for isolation. In order to expand the safety design of pyrotechnic devices and improve their safety and reliability, future development requires MEMS pyrotechnics with a partition self-recovery function, which means that the safety mechanism of the weapon system can autonomously recover and lock in a safe position after power failure. MEMS pyrotechnics with a partition self-recovery function play a key role in solving the safety issues of unexploded ordnance and other weapons and equipment. As a key component in weapon systems, MEMS pyrotechnics with recoverable partition functions will improve the overall safety and reliability of the weapon system.

### 3.2. Informationization

The informatization of MEMS S&A devices cannot be separated from the development of precision control technology and artificial intelligence technology. Through embedded systems and AI algorithms, self-diagnosis and predictive maintenance can be achieved, improving the overall automation level of the system [24,25,26,27,28,29,30,31,32,33].

#### 3.2.1. Precision of Information Perception and Control

The key to precise information perception lies in the development of micro-nano-sensor technology, and the key to precise control lies in optimizing control strategies. In the field of pyrotechnic security, the precise perception of small changes or potential threats is the foundation for ensuring safety. The development of micro-nano-sensor technology has brought revolutionary changes to the safety execution mechanism of pyrotechnic devices. Micro-nano-sensors, with their high precision, sensitivity, and miniaturization characteristics, provide core detection and sensing capabilities for MEMS S&A devices, becoming a key technology for achieving intelligent security.

During the storage, transportation, and use of pyrotechnic devices, they may be affected by various factors such as temperature, pressure, and vibration, which can pose a threat to the safety of pyrotechnic devices. Micro-nano-sensors can monitor these key parameters in real time, accurately capture any small changes, and provide real-time status information for safety S&A devices.

Specifically, the application of micro-nano-sensors in pyrotechnic safety S&A device is mainly reflected in the following aspects:

- Temperature monitoring: Pyrotechnics are highly sensitive to temperature changes, and excessively high temperatures may cause pyrotechnic failure or danger. Micro-nano-sensors can monitor the temperature of pyrotechnic devices in real time. Once an abnormal temperature is detected, an alarm is immediately triggered to remind relevant personnel to take appropriate measures.
- Pressure perception: Pyrotechnics may be subjected to compression or impact during transportation and storage, resulting in changes in internal pressure. Micro-nano-sensors can accurately sense these pressure changes, ensuring the safety of pyrotechnic devices during transportation and storage.
- Vibration detection: Vibration is also one of the important factors affecting the safety of pyrotechnic devices. Micro-nano-sensors can monitor the vibration of pyrotechnic devices in real time, analyze the source and intensity of vibration, and provide important reference information for safety mechanisms.

By integrating advanced algorithms and data processing capabilities, micro-nano-sensors can not only achieve autonomous detection and automatic recognition, but also provide real-time warnings. This enables the safety mechanism to detect potential threats in advance before danger occurs, improving the practicality of MEMS safety mechanisms and the safety of pyrotechnic devices.

The integration trend of micro-nano-sensor technology enables MEMS S&A devices to have higher integration and a smaller volume. With the continuous development of micro-nano-sensor technology, its volume is becoming smaller and its performance is becoming stronger. This enables security enforcement agencies to deploy more flexibly in various environments, whether it is narrow spaces or complex environments, to achieve effective monitoring and early warning.

The integration of micro-nano-sensor technology enables the S&A device to be more miniaturized and easy to install without sacrificing performance. This is very important for the miniaturization of pyrotechnic devices, as lightweight mounting mechanisms can be more easily installed on pyrotechnic devices, improving their safety and practicality.

Micro-nano-sensor technology provides core detection and sensing capabilities for pyrotechnic safety S&A devices, ensuring the safety of pyrotechnic devices during storage, transportation, and use by monitoring key parameters such as temperature, pressure, and vibration in real time. At the same time, the integration trend of micro-nano-sensor technology has enabled MEMS S&A devices to have higher integration and smaller volume, improving the applicability and practicality of MEMS S&A devices.

#### 3.2.2. Intelligent Information Judgment and Control Strategy

Research has aimed to develop higher-level information encryption and judgment technologies, adopt physical protection measures to prevent environmental signal misunderstanding, signal interference, and illegal operations, and ensure the safety of MEMS pyrotechnics.

Taking the MEMS S&A device driven by electric heating as an example, it mainly uses a MEMS electric heating S&A device to achieve active control of the partition, and the corresponding driving signals need to be generated jointly by sensors and logic control circuits. In future research work, differential-pressure sensors, absolute-pressure sensors, and acceleration sensors can be added to existing systems to measure flight speed, flight altitude, and flight acceleration information in simulated environments as a data basis for subsequent logical processing units. The logic processing unit processes and performs logical operations on the information collected by front-end sensors. When the environmental parameters sensed by the sensors meet the requirements for insurance release, the system outputs a specific driving signal. If the environmental parameters do not meet the conditions for insurance release, the logic processing system remains in a data waiting state until the sensor returns a signal that meets the logical conditions.

Taking the combination of a MEMS S&A device and artificial neural networks as an example, MEMS S&A devices play an important role in the field of pyrotechnic control due to their miniaturization, high precision, and fast response characteristics. Artificial neural networks can simulate the connection mode of human brain neurons, learn the inherent rules of data through training, and thus achieve the processing of complex problems. MEMS S&A devices can convert electrical signals into mechanical motion, achieving precise control at the microscale. The combination of the two is expected to provide new solutions for intelligent control in the micro-field of pyrotechnic devices. Specifically, by training neural network models, we can enable them to learn complex nonlinear relationships related to MEMS S&A device control. Once the model training is completed, control instructions can be generated in real time based on the input signals, and precise motion control can be achieved through MEMS S&A devices. This combination can not only improve the accuracy and efficiency of control, but also achieve adaptive control of complex environments.

**Figure 12:**
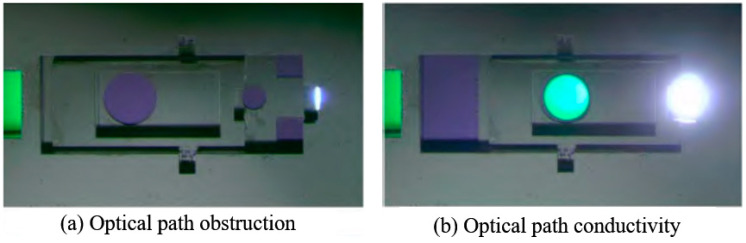
The optical feedback of MEMS SAD. Reprinted from Ref. [34].

As shown in Figure 12, Hu Tengjiang proposed a silicon-based SAD with state feedback function in order to obtain the real-time working status of the micro-safety and arming device (SAD) integrated into the MEMS initiation system [34]. The SAD is driven by the electro-thermal principle. Combined with the V-shaped electro-thermal actuator and planar pawl mechanism, the barrier structure in the SAD can be controlled actively. A light through the hole is designed on the barrier, and the real-time feedback of the motion status can be achieved by detecting the resistance changes of the photosensitive resistor.

The combination of artificial intelligence technology and MEMS S&A devices can not only improve the performance and safety of pyrotechnic devices, but also reduce production costs and improve production efficiency, providing strong support for the sustainable development of the pyrotechnic device field.

#### 3.2.3. Diversify Control Signals

Traditional control of MEMS S&A devices mainly relies on electrical signals, but with the development of technology, various new control signals adapted to more complex battlefield environments, such as microwave, laser, infrared, etc., are gradually being applied to the control of MEMS S&A devices. These new control signals have the advantages of strong anti-interference ability, fast transmission speed, and high safety, which can achieve more accurate and efficient control of MEMS S&A devices. For example, wireless remote control can be achieved using microwave signals, high-precision ranging and positioning can be achieved using laser signals, and human detection and motion tracking can be achieved using infrared signals.

**Figure 13:**
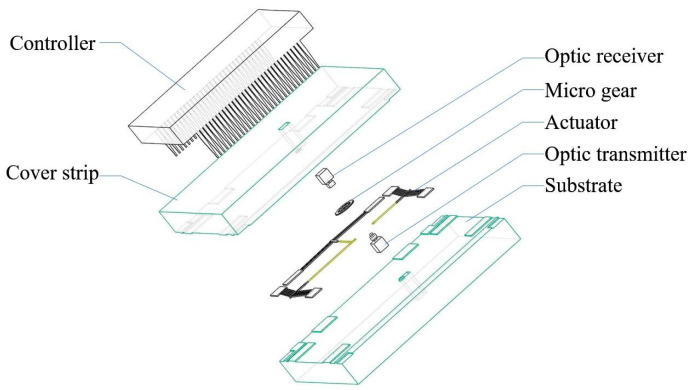
MEMS optical discriminator. Reprinted from Ref. [35].

As shown in Figure 13, Zhang Dongpeng proposed the manufacturing process of a set of MEMS optical combination lock discriminators which can be used in MEMS S&A devices [35]. Optical combination lock is a combination of mechanical combination lock and MEMS technology, which can effectively reduce the size of the combination lock. It not only guarantees the reliability and validity of password transmission, but also has the advantages of easy realization of password diversity and small structure size.

With the increasingly widespread application of wireless communication technology and new control signals in security mechanisms, their safety and reliability issues are becoming more prominent. In the future, it will be necessary to strengthen the security protection and reliability guarantee of wireless communication networks and new control signals to ensure the stable operation and data security of security systems.

#### 3.2.4. MEMS S&A Device Self-Energy Storage

The next step in integrating MEMS S&A devices with MEMS pyrotechnics is the fuse-level system integration of MEMS S&A devices. The fuse-level system integration of MEMS S&A devices is based on various sensors detecting environmental information and intelligent control of integrated circuits. Starting from MEMS processing and IC processing technology, the control circuit, MEMS S&A device, and energetic materials are integrated and designed and manufactured on one or more chips according to the the principle of process compatibility. The security system is highly integrated to form on-chip fuses, which are smaller in size and lighter in weight, suitable for mass production, reduce production costs, and improve reliability.

The foundation of the fuse-level system integration of MEMS S&A devices lies in the research of self-energy storage technology for MEMS S&A devices. The design integrates the transducer element, pyrotechnic agent, and S&A device in the pyrotechnic device, achieving the on-chip integration of the ignition energy in the S&A device and the partition mechanism. The system only needs to receive ignition command signals and does not need to receive ignition energy to achieve the ignition, detonation, and isolation of the device.

## 4. Conclusions

In the technological evolution of pyrotechnic devices, the application of MEMS S&A devices is gradually highlighting its important value, with a clear direction toward miniaturization, integration, and informatization. This technological innovation not only represents the cutting-edge trend in the field of pyrotechnics, but also is one of the key technologies to ensure the safety and reliability of pyrotechnics. Among them, the integrated design and manufacturing of MEMS S&A devices and MEMS pyrotechnics have become the core direction for the future development of the pyrotechnic device field.

Through integrated design, MEMS S&A devices can be cleverly embedded into MEMS pyrotechnics, forming a highly integrated micro-explosive train, enabling pyrotechnic devices to have more precise and efficient control capabilities at the microscale. By actively controlling various signals such as electrical signals and environmental force signals, it is possible to accurately achieve different actions of the safety mechanism, thereby ensuring precise isolation and alignment of the micro explosive train. This control mechanism not only improves the performance of pyrotechnic devices, but also achieves refined control of explosive trains at the system level.

In summary, MEMS S&A devices have shown great potential and value in the field of pyrotechnic devices. The characteristics of miniaturization, integration, and informatization have significantly improved the safety and reliability of pyrotechnic devices. In the future, with the continuous advancement and innovation of technology, the integrated design and manufacturing of MEMS S&A devices and MEMS pyrotechnics will lead the pyrotechnic field toward a safer, more reliable, and intelligent future.

## Figures and Tables

**Table 1:** Classification of MEMS S&A devices.

Classification Method	Name Title 2	Characteristics
Explosion sequence	Displaced explosive train structure	Reliable explosion-proof/explosion transmission, high assembly accuracy requirements
	Isolated explosive train structure	Small device size and high level of intelligence
Manufacturing materials	Silicon-based	Small size, good compatibility with MEMS technology
	Non silicon-based	High reliability and structural strength
Driving method	Environmental forces drive	During the launch process, recoil and centrifugal force
	Gunpowder power drive	Fireworks chemicals produce high-temperature and high-pressure gases
	Electric thermal drive	Joule heating effect and thermal expansion of materials
	Other forces drive	Electromagnetic, piezoelectric, shape memory alloy, etc.
	Multi-principle joint drive	Mechanical environmental forces and other driving methods jointly drive
